# Mitochondrial respiratory dysfunctions of blood mononuclear cells link with cardiac disturbance in patients with early-stage heart failure

**DOI:** 10.1038/srep10229

**Published:** 2015-05-28

**Authors:** Peng Li, Bin Wang, Fang Sun, Yingsha Li, Qiang Li, Hongmei Lang, Zhigang Zhao, Peng Gao, Yu Zhao, Qianhui Shang, Daoyan Liu, Zhiming Zhu

**Affiliations:** 1 Center for Hypertension and Metabolic Diseases, Department of Hypertension and Endocrinology, Daping Hospital, Third Military Medical University, Chongqing Institute of Hypertension, Chongqing 400042, China; 2 Department of Cardiology, Institute of Clinical Medicine of Zunyi Medical College, Affiliated Hospital of Zunyi Medical College, Zunyi, Guizhou 563003, China

## Abstract

Patients with cardiometabolic risk factors and asymptomatic cardiac hypertrophy are hallmarks of early-stage heart failure (HF). We hypothesized that mitochondrial respiratory dysfunctions of peripheral blood mononuclear cells (PBMCs) may be associated with inflammation and oxidative stress in early-stage HF patients complicated with cardiometabolic risk factors. Totally 49 subjects were enrolled with 25 early-stage HF patients (stages A and B) having cardiac hypertrophy and dysfunction and 24 healthy controls. It showed that excessive inflammation and reduced antioxidant capacity were closely associated with cardiac abnormalities in early-stage HF patients. Furthermore, the values of mitochondrial respiratory functional parameters R, CI_OXPHOS_, CII_OXPHOS_, CI+II_OXPHOS,_ CI+II_ETS_ and CII_ETS_ were significantly lowered in early-stage HF patients. Interestingly, these respiratory parameters were correlated with inflammation and antioxidant capacity in participants. Finally, cardiometabolic risk factors such as salt intake and blood pressure were related to the mitochondrial respiratory dysfunctions, which were further validated by *in vitro* experiments. Our study indicated that cardiometabolic risk factor-mediated mitochondrial respiratory dysfunctions of PBMCs link with the cellular inflammation / oxidative stress and cardiac disturbance in early-stage HF.

Heart failure (HF) is becoming a chronic disease that severely threatens the public health, with over 23 million people affected worldwide^1^. Clinically, the process of HF can be divided into four stages: A, B, C and D. Stage A is characterized by presence of HF risk factors, but is asymptomatic and without evidence of cardiac remodeling or impaired cardiac functions. Stage B is still asymptomatic but with left ventricular (LV) hypertrophy or impaired LV function. In general, stage A and stage B HF are considered as early-stage HF and important periods for HF prevention and treatment^2^. Cardiometabolic risk factors such as dyslipidemia, hypertension, obesity, diabetes mellitus, and high salt intake contribute to the development of early-stage HF^3^. However, the relationship between these cardiometabolic risk factors and early-stage HF are not fully understood^1^.

Clinical and experimental evidence shows that oxidative stress and chronic inflammation are important initiators for the development of HF^4^. Cardiometabolic risk factors cause chronic inflammation and reactive oxygen species (ROS)^5^,^6^,^7^, which are closely related to HF, but the source of this chronic inflammation and oxidative stress remains elusive. Peripheral blood mononuclear cells (PBMCs) are a group of mononuclear immune cells that exist in blood and exhibit important roles in the immune responses of the organism^8^. The mitochondrial dysfunctions of PBMCs may be responsible for the production of inflammation and ROS^9^. Therefore, it warrants further elucidating the role of mitochondrial dysfunction of PBMCs in patients with early-stage HF.

In the present study, we examined the associations between cardiometabolic risk factors and mitochondrial respiratory functions of PBMCs and investigated the relationship between mitochondrial respiratory dysfunctions and inflammation / oxidative stress as well as cardiac disturbance in patients with early-stage HF.

## Results

### Baseline characteristics of participants

The baseline characteristics of the recruited participants are listed in Table 1. Totally 49 subjects were recruited into our study, with 25 early-stage HF patients and 24 normal controls. Among the 25 patients, 14 patients were diagnosed with essential hypertension (EH), 4 patients were diagnosed with T2DM and 7 patients were with both EH and T2DM. There was no difference in mean age or gender ratio between normal controls and patients with early-stage HF (*P* > 0.05). However, the values of blood pressure, fasting blood glucose (FBG) and plasma lipids were significantly higher in patients with the early-stage HF than normal controls (*P* < 0.05-0.01). In addition, 24 h urinary sodium excretion and dietary salt intake were higher in patients with early-stage HF than normal controls (*P* < 0.05). Although their 24 h urinary potassium excretion trended to be lower, this difference was not significant. These results indicate that patients with early-stage HF are complicated with multiple cardiometabolic risk factors.

### Cardiac hypertrophy and dysfunction in early-stage HF

Table 2 showed that, in addition to left ventricular diameter (LVD), the values of interventricular septum thickness (IVST) and left ventricular posterior wall thickness (IVWPT) were higher in the patients with early-stage HF than normal controls (*P* < 0.0001 and *P* < 0.05, respectively). The E/A ratio was significantly lower in the patients with early-stage HF than normal controls (*P* < 0.01). The patients’ ejection fraction (EF) (%) and fractional shortening (FS) (%) values trended to be lower but did not reach significance. These data indicate that patients with early-stage HF have cardiac remodeling and minor dysfunctions.

### Relationship between cardiac disturbance and cardiometabolic risk factors

**Figure 1:**
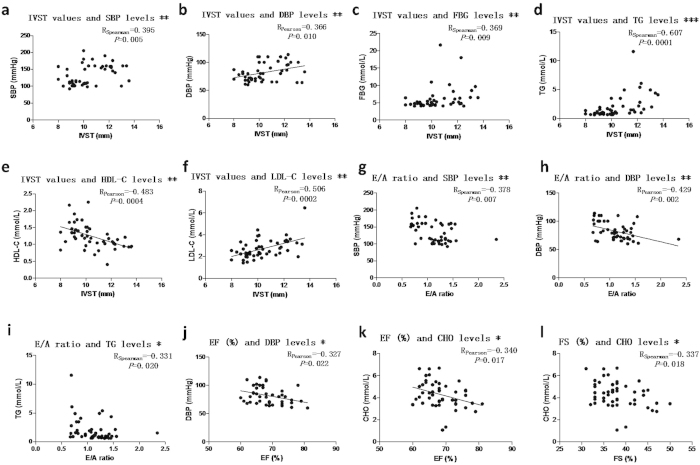
Relationship between cardiac parameters and cardiometabolic risk factors. For those with statistically significant correlations, scatter diagrams were shown, and then correlation coefficient R values and *P* values were listed. *: *P* < 0.05, **: *P* < 0.01, ***: *P* < 0.0001.

Firstly, we analyzed whether the parameters above followed normal distribution. Apart from FS (%), SBP, FBG and triglyceride (TG), other parameters all followed normal distribution. As shown in Fig. 1, the values of IVST were closely correlated with cardiometabolic risk factors, such as blood pressure, FBG, TG, high density lipoprotein cholesterol (HDL-C) and low density lipoprotein cholesterol (LDL-C) in the participants. Meanwhile, cardiac functional parameters such as E/A, EF (%) and FS (%) values were correlated with blood pressure and/or plasma lipid levels in the participants. These data indicate that cardiometabolic risk factors may contribute to cardiac abnormality in early-stage HF.

### Increased inflammation and reduced antioxidant capacity in early-stage HF

**Figure 2:**
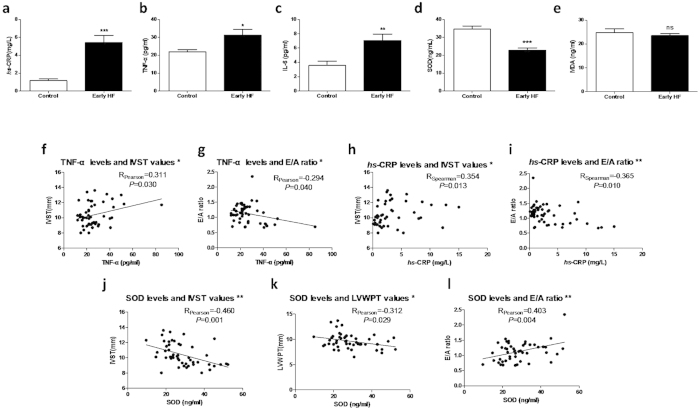
Increased inflammations and decreased antioxidant capacity are associated with cardiac lesions. (**a** to **e**)The plasma levels of inflammatory factors *hs*-CRP, IL-6, TNF-α and ROS parameters SOD, MDA were measured by ELISA. (**f** to **l**) The associations between inflammatory and oxidative stress factors verses cardiac parameters were calculated. Correlations with significant differences were shown as (**a** to **e**). *: *P* < 0.05, **: *P* < 0.01, ***: *P* < 0.0001.

To examine the changes to inflammation and oxidative stress in early-stage HF, several inflammatory factors and the oxidative stress parameters were measured in plasma. As shown in Fig. 2a-2c, plasma high-sensitivity C-reactive protein (*hs*-CRP), IL-6 and TNF-α were significantly higher in the patients with early-stage HF than normal controls. Meanwhile, plasma superoxide dismutase (SOD), an index for antioxidant capacity, was significantly reduced but plasma malondialdehyde (MDA) remained unchanged in patients with early-stage HF (Figs. 2d-2e). These results suggest that excessive inflammation and reduced antioxidant capacity occur in early-stage HF patients.

### Oxidative stress and inflammatory factors are associated with cardiac lesions

Before conducting bivariate correlations, normal distribution was tested in the inflammatory and oxidative stress parameters. It showed that IL-6 and *hs*-CRP did not follow normal distribution. As shown in Fig. 2f–2l, plasma TNF-α and *hs*-CRP were correlated with IVST and the E/A ratio in the participants. Meanwhile, SOD values were correlated with IVST, LVWPT and the E/A ratio. These results further support the notion that inflammation and oxidative stress are involved in the cardiac lesions in early-stage HF patients.

### Mitochondrial respiratory dysfunctions of PBMCs in early-stage HF

**Figure 3:**
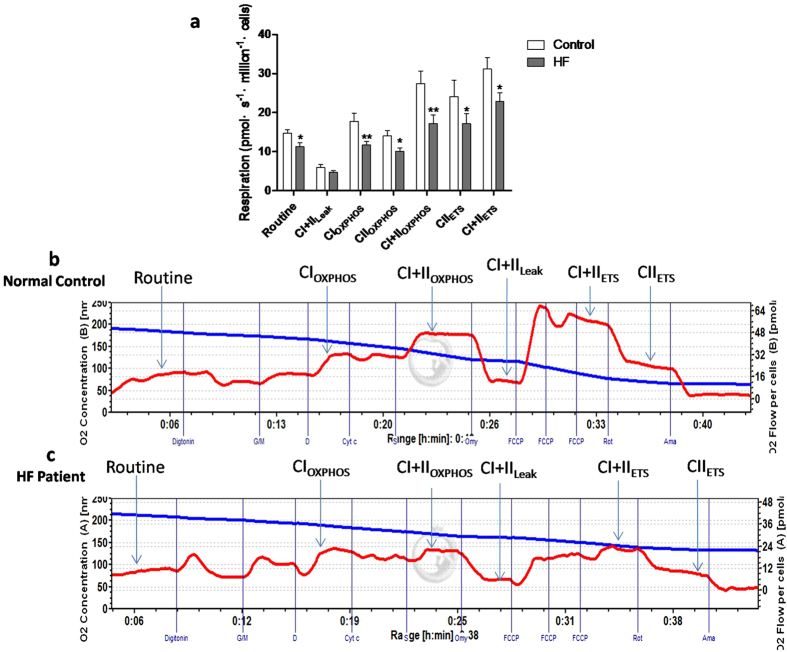
Mitochondrial respiratory dysfunctions of PBMCs in early-stage HF. (**a**) Oxygen consumptions at different mitochondrial stages of PBMCs were measured by Oxygraph-2 k high-resolution respirometry. Routine values, respiration in the original state; CI+II_Leak_, respiration on CI+II substrates to compensate for proton leak; CI_OXPHOS_, CI-dependent oxidative phosphorylation; CII_OXPHOS_, CII-dependent oxidative phosphorylation; CI+II_OXPHOS_, oxidative phosphorylation providing CI and CII substrates; CI+II_ETS_, non-coupled respiration with CI and CII substrates, is considered as maximum capacity of the ETS state; CII_ETS_, non-coupled CII-dependent respiration. (**b**, **c**) Typical graphs of high-resolution respirometry of PBMCs from healthy volunteers (**b**) and early-stage HF patients (**c**). Red line represents oxygen consumption, while blue line means oxygraph decreasing traces of oxygen concentration. *: *P* < 0.05, **: *P* < 0.01.

Because of the importance of mitochondrial respiratory function in influencing the function of PBMCs, mitochondrial respiratory functional parameters, namely routine respiratory value (R value), CI+II_LEAK_ (respiration on CI+II substrates to compensate for proton leak), CI_OXPHOS_ (Complex I-dependent oxidative phosphorylation), CII_OXPHOS_ (Complex II-dependent oxidative phosphorylation), CI+II_OXPHOS_ (oxidative phosphorylation providing CI and CII substrates), CII_ETS_ (non-coupled CII-dependent respiration) and CI+II_ETS_ (non-coupled respiration with CI and CII substrates), were measured by high-resolution respirometry (Oxygraph-2 k; Oroboros Instruments, Austria). As shown in Fig. 3a, apart from CI+II_LEAK_, all the functional parameters of mitochondrial oxygen consumption were significantly reduced in patients with early-stage HF compared with normal controls, indicating reduced mitochondrial respiratory functions. Fig. 3b,3c show representative tracings for mitochondrial respiratory function in normal controls and early-stage HF patients, respectively, showing an obvious disorder of cellular mitochondrial function in early-stage HF patients. These data suggest that mitochondrial respiratory dysfunction of peripheral blood cells occurs in early-stage HF.

### Correlation between mitochondrial respiratory function of PBMCs and oxidative stress/inflammation

**Figure 4:**
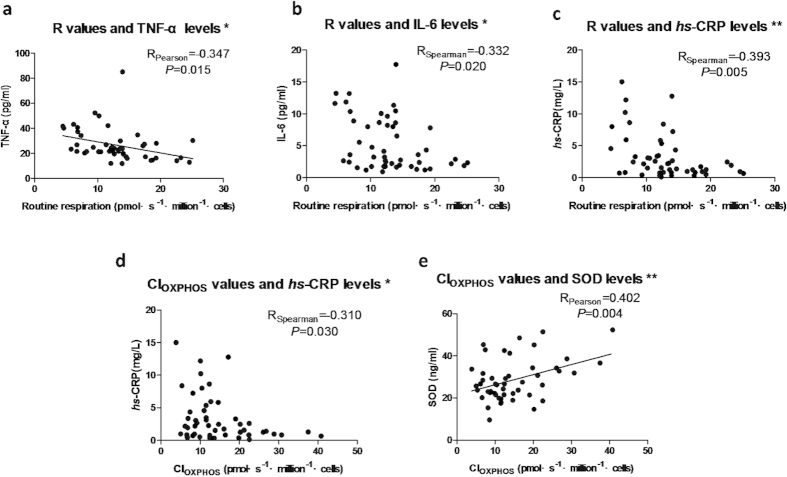
Correlation between mitochondrial respiratory functions of PBMCs and oxidative stress/inflammation. Associations with statistical significance were shown in this figure. *: *P* < 0.05, **: *P* < 0.01.

To test whether reduced mitochondrial respiratory functions of PBMCs link with oxidative stress / inflammation, bivariate correlations were performed. Mitochondrial respiratory parameter R value was negatively correlated with TNF-α, IL-6 and *hs*-CRP (Figs. 4a–4c), indicating that upregulation of inflammation was closely correlated with reduction of R values. Mitochondrial CI_OXPHOS_ was correlated with *hs*-CRP and SOD (Figs. 4d,4e). These results indicate that functional status of mitochondrial respiratory capacity, especially the CI_OXPHOS_ capacity, was associated with the production of inflammation and oxidative stress in PBMCs.

### Cardiometabolic risk factors are closely correlated with mitochondrial respiratory function of PBMCs

**Figure 5:**
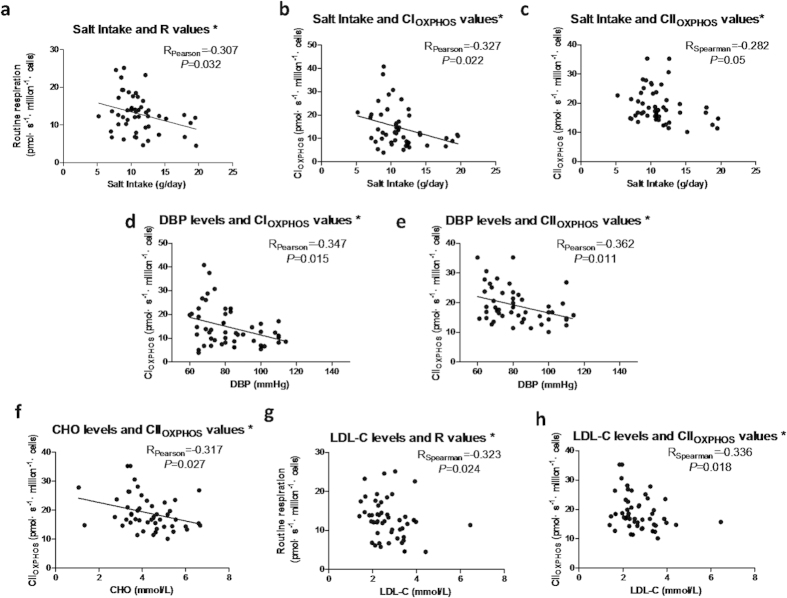
Cardiometabolic risk factors are closely correlated with mitochondrial respiratory function of PBMCs. Relationships between salt intake and mitochondrial respiratory functions (**a**, **b** and **c**), between blood pressure and mitochondrial respiratory functions (**d**, **e**), between plasma lipids and mitochondrial respiratory functions (**f**, **g** and **h**). Correlations with significant difference were listed. *: *P* < 0.05.

We further examined whether there were correlations between the changes to mitochondrial respiratory function and cardiometabolic risk factors. It showed that dietary salt intake was inversely correlated with mitochondrial R, CI_OXPHOS_ and CII_OXPHOS_ (Figs. 5a–5c). Meanwhile, DBP and plasma lipids parameters were negatively correlated with CI_OXPHOS_ and CII_OXPHOS_ (Figs. 5d–5h). These results indicate that cardiometabolic risk factors are closely correlated with the mitochondrial respiratory dysfunctions in early-stage HF.

### Effect of sodium chloride on the cellular mitochondrial respiratory function of human THP-1 cells

**Figure 6:**
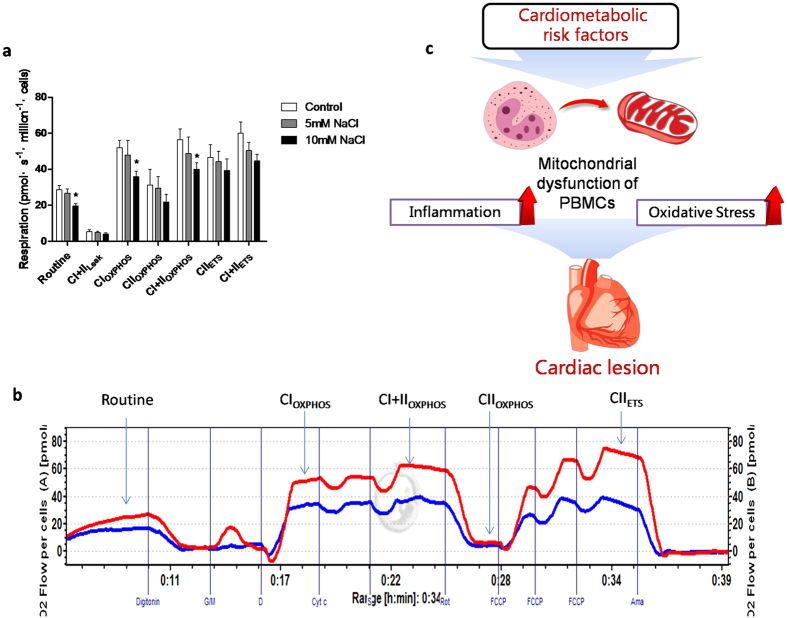
Effect of sodium chloride on the cellular mitochondrial function of THP-1 cells. (**a**) High NaCl solution was made by adding additional 5 mM and 10 mM NaCl to the solutions, and mannitol was used to balance osmotic pressures. Cells were incubated in the solutions for 24 h, and then mitochondrial oxygen consumptions of THP-1 cells with or without NaCl treatment were measured by high-resolution respirometry. (**b**) Typical graph of high-resolution respirometry of THP-1 cells with10 mM NaCl treatment (Blue line) and without NaCl treatment. (**c**) Schematic illustration for the relationship between cardiometabolic risk factors, mitochondrial respiratory dysfunctions of PBMCs, inflammation / oxidative stress and cardiac lesions in early-stage heart failure.*: *P* < 0.05, **: *P* < 0.01.

To further validate whether metabolic risk factor can directly impair mitochondrial respiratory function, THP-1 human monocytes were used to test the effect of high concentration NaCl on mitochondrial respiratory functions. Our results clearly demonstrated that administration of 10 mM, but not 5 mM, NaCl into the cell culture medium significantly inhibited the mitochondrial respiratory functions, particularly CI_OXPHOS_ (Fig. 6).

## Discussion

The present findings provide the first evidence for a peripheral cellular mitochondrial respiratory dysfunction in patients with early-stage HF. The major findings of this study are that early-stage HF patients complicated with multiple cardiometabolic risk factors were associated with cardiac hypertrophy and dysfunction. Compared with normal control participants, excessive inflammation and reduced antioxidant capacity as well as mitochondrial dysfunction of PBMCs appeared in early-stage HF patients. Furthermore, mitochondrial respiratory function of PBMCs was correlated with inflammation and oxidative stress as well as cardiac disturbance and several cardiometabolic risk (Fig. 6c).

High blood pressure, high-salt diet, diabetes, dyslipidemia and obesity are major risk factors for HF^1^,^10^,^11^,^12^,^13^,^14^. These cardiometabolic risk factors may lead to cardiac hypertrophy and dysfunction^15^. In the present study, patients with early-stage HF had higher blood pressure, increased fasting blood glucose and lipids, and higher salt intake compared with control participants. These cardiometabolic risk factors are associated with changes in cardiac structure and function. However, the relationship between cardiometabolic risk factors and cardiac lesions remain to be defined.

Excessive ROS and inflammation occur in patients with hypertension, obesity, T2DM and dyslipidemia^16^. Furthermore, there is an inverse relationship between plasma antioxidants or total antioxidant capacity and the metabolic syndrome^17^,^18^. Our study showed that plasma levels of inflammatory factors were significantly higher but SOD significantly lower in patients with early-stage HF than that in normal controls. In addition, plasma levels of inflammatory factors and SOD were closely correlated with cardiac disturbance in the participants. Oxidative stress can trigger inflammation and cardiovascular lesions^19^. This study indicates that persistent inflammation and impaired antioxidant capacity might be associated with the pathogenesis of HF.

There are multiple sources of ROS and inflammation^20^. However, activation of immune-competent cells, leading to local and finally systemic inflammation and the associated status of oxidative stress are central events^4^,^21^. Monocyte/macrophage accumulation at the lesion is a key factor in the inflamed cardiovascular tissues^19^,^22^. Mitochondria are often regarded as the cellular power houses in the control of cardiac energy metabolism through their ability to generate ATP^9^. ATP in mitochondria is generated by the electron transport chain (ETC), which is composed of four oxidoreductase complexes (complexes I, II, III, and IV) and ATP synthase (complex V). The functions of a single complex can be measured by high-resolution respirometry, which measures the values of CI_OXPHOS_, CII_OXPHOS_, CI+II_OXPHOS,_ CI+II_Leak_, CI+II_ETS_ and CII_ETS_ after administration of inhibitors or activators of these complexes^23^. Defects of the electron transport chain, specifically at complex I, may be responsible for cardiac hypertrophy and even HF^24^.

We showed that all functional parameters of mitochondrial oxygen consumption especially complex I and II phosphorylating activities, were significantly reduced in patients with early-stage HF compared with normal controls. The possible reasons for these electron transport reductions were reduced biogenesis of mitochondria or increased mitophagy per mononuclear cell^25^. It could also reflect impaired substrate entry or oxidation proximal to the ETC. In addition to regulating cardiac energy metabolism, cellular mitochondrial dysfunction can directly promote cell death, inflammation, and oxidative stress^17^. Monocytes/macrophages play important roles in the progression of cardiovascular diseases^22^. Our previous work has demonstrated that hypertension is correlated with increased activation and migration of monocytes^26^,^27^. Furthermore, cellular mitochondrial ROS production could influence the expressions of pro-inflammatory cytokines and immune modulators^28^,^29^. Similarly, Kong *et al.*^30^ reported that the mitochondrial transmembrane potential (MTP) of lymphocytes was significantly reduced and oxidants were increased in patients with chronic heart failure, which were correlated with clinical features and immune abnormalities. Our study also demonstrated that the mitochondrial respiratory functions of PBMCs in the participants were associated with the oxidative stress and inflammation.

Cardiometabolic risk factors such as high glucose can cause mitochondrial lesions^31^, but the causality between risk factors and mitochondrial respiratory dysfunction is unclear. In this study, salt intake and blood pressure are associated with the mitochondrial respiratory dysfunction in the participants. We further confirmed that increased sodium concentration inhibited mitochondrial respiratory functions, particularly R value, complex I and II oxidative phosphorylation capacity. This result indicates that cardiometabolic risk factors might directly jeopardize the cellular mitochondrial respiratory functions of PBMCs, which may link with inflammation / oxidative stress in the early-stage HF patients with multiple cardiometabolic risk factors.

There are several limitations in this study. Firstly, this study is a cross-sectional study and has a relatively small size of participants. Thus, we cannot draw causality conclusion about relationship between mitochondrial respiratory functions, inflammatory / oxidative stress and cardiac lesions without longitudinal follow-up. Secondly, patients included in this study had both cardiac lesions and metabolic disorders. Mitochondrial respiratory dysfunctions and inflammatory / oxidative stress are related to metabolic disturbance and/or cardiac lesions. Thus, we acknowledged the comorbidities existed in our studied patients. Thirdly, other factors such as medicine administration in patients may also influence the results.

In summary, we have demonstrated for first time that mitochondrial respiratory dysfunctions of PBMCs may link with cardiometabolic risk factors and cardiac lesions in patients with early-stage HF. Excessive inflammation and oxidative stress are associated with cardiac abnormalities. Cardiometabolic risk factor-mediated mitochondrial respiratory dysfunctions of PBMCs are involved in the cellular inflammation and oxidative stress in early-stage HF. Our findings provide novel insight into the role of mitochondrial respiratory dysfunctions in the pathogenesis of HF. Therefore, targeting the mitochondrial dysfunctions of PBMCs may be a promising intervention to prevent early-stage HF development.

## Methods

### Study participants

Adult participants (40-65 years old) were recruited to our center in Daping Hospital in Chongqing, China. The criteria for early-stage HF were as follows: 1) Patients were in stage A or B of HF according to the guidelines of ACCF/AHA. 2) Patients were diagnosed with EH, T2DM or both. Hypertension was defined according to the guidelines of WHO/ISH^32^. The measurement protocol of BP was previously described^33^. T2DM was defined as FBG ≥ 126 mg/dl or HA1c > 6.5% or ongoing treatment for T2DM. 3) Patients were not pregnant and had no other major defects in other systems or organs. Healthy volunteers were screened with qualified medical examinations in our hospital. They were taking no medications during this period and had normal BP and FBG. Blood samples of participants were obtained in the morning by venipuncture. Blood glucose and lipid parameters were measured in the Chongqing Institute of Hypertension. The study protocol was approved by the ethics committee of the third affiliated hospital to the Third Military Medical University. All participants have signed the written informed consent.

### Echocardiography

The cardiac function and the structural parameters were detected by ultrasonic cardiogram using the Ultrosound Sonos 7500 (Koninklijke Philips Electronics N.V, The Netherlands) with a 1.6/3.2 MHz transducer. Both M-mode and two-dimentional measurements were performed according to the methods recommended by the American Society of Echocardiography. The main parameters include IVST, LVWPT, LVD, E/A ratio, EF and FS. All the participants were examined by the same echocardiography to reduce the systematic error within the group.

### Measurement of inflammatory factors, SOD and MDA levels in the plasma by ELISA

ELISA was used to detect the expression levels of IL-6 (EK0410, Boster Biological Technology, Wuhan, China), TNF-α (EK0525, Boster Biological Technology, Wuhan, China), SOD (SES134Hu, Cloud-clone Corp, Houston, TX, USA) and MDA (CEA597Ge, Cloud-clone Corp, Houston, TX, USA) in the plasma, according to the protocols of the kits.

Plasma samples were collected and centrifuged at the speed of 1000 rpm for 20 min. Then 100 μl supernatant was added to the provided 96-well plate for 2h in 37 °C while standard curve was made in the same plate. A hundred milliliters diluted primary antibody was added to each well and the plate was incubated in 37 °C for 1h. Afterwards, each well was washed with wash buffer or 0.01 M PBS for 3 times. Then 100 μl diluted secondary antibody was then added and incubated in 37 °C for 30 min. The plate was then washed for 5 times in the same way. At last, reaction substrate and stop buffer was in turn added and the OD450 values were measured with the multiskan microplate reader.

### Isolation of PBMCs

Heparinized blood was collected via venipuncture from patients and then 10 ml blood samples were centrifuged at 1500 rpm for 5 min at room temperature. Then cells layer of venous blood was mixed with the same volume of HBSS (Gibco, NY, USA). The mixture of each sample was added with 1.5 ml Histopaque solution (Sigma, 10771, St. Louis, MO, USA) and was centrifuged at 2300 rpm for 15 min at room temperature. Cell layers were then collected and transferred to one volume of HBSS to wash the cells. The mixture was then centrifuged at 2500 rpm for 5 min at room temperature, and the pellet was resuspended by mitochondrial respiration medium (MiR05), then cells were used for detections of mitochondrial respiration.

### Cell culture and treatment

Human monocytic cell line THP-1 was purchased from Type Culture Collection of the Chinese Academy of Sciences (Shanghai, China) and passaged for less than six months in our laboratory. Cells were maintained in 1640 medium with 10% FBS (Gibco, NY, USA), 100 U/ml penicillin and 100 μg/ml streptomycin (Hyclone, Logan, Utah, USA) added at 37 °C in a humidified incubator containing 5% CO2. After resuscitation and passed for three passages, cells entered exponential phase and were planted to 6-well plate with the concentration of 1 × 10^6 ^cells/ml. Then cells were treated with different concentrations of NaCl (5 mM and 10 mM) for 24 h and mannitol was used to equivalent the osmotic pressure, while control group was treated with 15 mM mannitol only. After 24 h, cells were harvested for detections of mitochondrial respiratory functions. Meanwhile, trypan blue staining was performed to check the cultured cell viability.

### High-resolution respirometry

Respiratory function was analyzed in a two-channel titration injection respirometer (Oxygraph-2 k; Oroboros Instruments, Innsbruck, Austria) equipped with two chambers at 37 °C. PBMCs or THP-1 cells were harvested, suspended in MiR05 solution, and transferred separately to oxygraph chambers at a final cell density of approximately 2 × 10^6^ (PBMCs) or 1 × 10^6^ (THP-1 cells) per milliliter.

To analyze mitochondrial respiratory function in detail, two complex protocols applying substrates, uncouplers, and inhibitors were used. Routine respiration (R value) was measured while the respiration was stabilized. Then, the plasma membrane was permeabilized with digitonin (10 μg per 10^6^ cells). CI_OXPHOS_ was measured after adding glutamate (G, 5 μM), malate (M, 2 mM), and ADP (D, 5 mM). The integrity of the outer mitochondrial membrane was checked by the addition of cytochrome *c* (Cytc, 10 μM). Sequentially, succinate (S, 10 mM) was added to induce maximal OXPHOS capacity with convergent input through CI+II_OXPHOS_. In the first protocol, oligomycin (Omy, 2 μg/ml) was added to inhibit ATP synthase and induce LEAK respiration (CI+II_Leak_). Maximal convergent respiratory capacity of the ETS was subsequently obtained by titrating FCCP (injected stepwise up to 1-1.5 μM) (CI+II_ETS_). Next, rotenone (0.5 μM) addition allowed the determination of CII-supported non-coupled respiration (CII_ETS_). Residual oxygen consumption (ROX) was determined by adding antimycin-A (2.5 mM), a complex III inhibitor. In the second protocol, addition of rotenone (Rot, 0.5 μM) allowed the determination of CII_OXPHOS_. After uncoupling with FCCP (injected stepwise up to 1-1.5 μM) in the non-coupled state, CII_ETS_ was measured. Residual oxygen consumption (ROX) was evaluated after the inhibition of CIII with antimycin A (Ama, 2.5 μM).

### Statistical analysis

Clinical characteristics and echocardiology parameters of early-stage HF patients and healthy volunteers were analyzed using the unpaired t-test or Wilcoxon signed rank test for continuous variables and chi-squared test for categorical variables, respectively. Group differences comparing the values of inflammatory factors, oxidative stress parameters and mitochondrial oxygen consumption in the two groups were analyzed by the unpaired t-test or nonparametric rank-sum test to calculate, depending on whether these variables followed a normal distribution. Pearson’s linear regression analysis and Spearman’s nonparametric correlation analysis were used to determine the relationships between risk factors, mitochondrial oxygen consumption, oxidative stress values, inflammatory parameters and cardiac remodeling parameters. *P* < 0.05 was considered to be statistically significant. Data were expressed as Mean±SEM and analyzed by SPSS 18.0.

## Additional Information

**How to cite this article**: Li, P. *et al.* Mitochondrial respiratory dysfunctions of blood mononuclear cells link with cardiac disturbance in patients with early-stage heart failure. *Sci. Rep.* **5**, 10229; doi: 10.1038/srep10229 (2015).

## Figures and Tables

**Table 1:** Baseline characteristics of the participants.

**Variable**	**Normal Controls (n = 24)**	**Early stage HF (n = 25)**	***P*** **value**
Age (Years)	47±3	49±3	0.6397
Male (%)	46	48	0.8888
FBG (mmol/L)	4.64±0.10	7.38±0.85**	0.0029
SBP (mmHg)	109±2	151±5***	<0.0001
DBP (mmHg)	72±1	91±3***	<0.0001
TG (mmol/L)	0.93±0.07	2.90±0.48***	0.0002
CHO (mmol/L)	3.95±0.13	4.61±0.30	0.0525
HDL-C (mmol/L)	1.47±0.06	1.03±0.05***	<0.0001
LDL-C (mmol/L)	2.19±0.09	3.23±0.19***	<0.0001
Salt Intake (g/day)	10.12±0.53	12.12±0.79*	0.0425
Urine Na^+^ (mmol/L)	172.13±9.03	206.06±13.47*	0.0435
Urine K^+^ (mmol/L)	43.88±3.12	37.56±2.07	0.0955

1. Abbreviations: fasting blood glucose (FBG), systolic blood pressure (SBP), diastolic blood pressure (DBP), triglyceride (TG), cholesterol (CHO), high density lipoprotein cholesterol (HDL-C), low density lipoprotein cholesterol (LDL-C).

2. *: *P* < 0.05; **: *P* < 0.01; ***: *P* < 0.0001.

**Table 2:** Parameters of Echocardiography.

**Variable**	**Normal Controls (n = 24)**	**Early stage HF (n = 25)**	***P*****value**
IVST (mm)	9.38±0.13	11.38±0.28***	<0.0001
LVWPT (mm)	9.10±0.24	10.15±0.32*	0.0121
LVD (mm)	42.83±0.65	44.32±0.76	0.1444
E/A ratio	1.29±0.05	0.98±0.06**	0.0003
EF (%)	69.63±0.99	67.40±0.83	0.0899
FS (%)	39.25±1.36	37.28±0.81	0.2151

1. Abbreviations: interventricular septum thickness (IVST), left ventricular posterior wall thickness (LVWPT), left ventricular diameter (LVD), ejection fraction (EF) and fractional shortening (FS).

2. *: *P* < 0.05; **: *P* < 0.01; ***: *P* < 0.0001.
